# Non-arteritic Anterior Ischemic Optic Neuropathy Sequela From Potential COVID-19-Associated Coagulopathy

**DOI:** 10.7759/cureus.52158

**Published:** 2024-01-12

**Authors:** Sanad Naber, Nadia Alinaghizadeh, Amy Kotecha

**Affiliations:** 1 Department of Research and Scholarly Activity, Rocky Vista University College of Osteopathic Medicine, Ivins, USA; 2 Opthalmology, Capital Vision Eye and Surgical Center, Arlington, USA

**Keywords:** hypercoagulable state, sudden vision loss, covid-19, ischemic optic neuropathy, non-arteritic anterior ischemic optic neuropathy (naion)

## Abstract

We report a case involving a post-menopausal female who experienced a sudden loss of peripheral vision in her right eye seven months after a confirmed COVID-19 infection. MRI scans of the brain and orbit excluded neuritis and multiple sclerosis, leading to the diagnosis of non-arteritic anterior ischemic optic neuropathy (NAION). It is known that the intense inflammatory condition resulting from acute respiratory distress syndrome triggered by SARS-CoV-2 infections can result in a heightened tendency for blood clot formation. Emerging research underscores the potential link between the likelihood of a thrombotic event in the eye as a consequence of COVID-19 infection and the development of NAION. The connection between NAION and COVID-19, whether it is correlative or coincidental, remains uncertain. However, this case report aims to present evidence for the plausibility of this link and offer insights into potential ophthalmologic complications caused by COVID-19.

## Introduction

Non-arteritic anterior ischemic optic neuropathy (NAION) is commonly attributed to microcirculatory insufficiency within the optic nerve head and subsequently causes unilateral vision loss [1]. However, the precise nature of the vasculopathy and its exact location remain uncertain [2-4]. Recent studies are beginning to shed light on specific vascular risk factors, such as diabetes, hypertension, and hyperlipidemia, which may elevate the susceptibility to NAION development [5,6]. Additionally, investigators are delving into the influence of prothrombotic risk factors on the pathogenesis of this condition [3].

The acute respiratory distress syndrome caused by SARS-CoV-2 infection can lead to a severe inflammatory state and endothelial damage, resulting in hypercoagulable states [7-9]. Numerous retrospective studies have described a prothrombotic condition in many patients with COVID-19 [10-12]. This hypercoagulable state likely contributes to an increased risk of developing NAION in some COVID-19 patients [13,14]. This clinicopathological case report describes a post-menopausal patient who experienced sudden, painless inferotemporal visual field loss after a COVID-19 infection.

## Case presentation

A post-menopausal patient with no history of diabetes or hypertension presented with a sudden, painless decrease in the peripheral vision of her right eye seven months after the onset of COVID-19 symptoms. She initially noted the visual changes after she fell due to the altered perception. The patient’s past medical history included menopause, tinnitus, alopecia, anemia, sinus lift procedure, gastric bypass with secondary calcium and iron deficiency, vitamin D deficiency, and osteoporosis. Her past ocular surgical history included LASIK surgery in both eyes in 2000.

After the fall, the patient was initially seen by her optometrist for ocular symptoms, and a small Drance hemorrhage was suspected. The patient was then referred to us for consultation. She reported experiencing flu-like symptoms seven months ago following her return from her sinus lift procedure in Cancun, Mexico. Initial symptoms included elevated temperature, nausea, persistent headaches, cough, chest tightness, and pain. The patient reported that her symptoms worsened three days later, describing it as “the worst flu” she has ever experienced. She went to visit her primary care physician and was prescribed Theraflu. One month after her initial symptoms, she developed a strong persistent cough, which lasted seven weeks. No other symptoms were noted. Five months after her initial symptoms, a SARS-CoV-2 antibody test came back with positive results. She later participated in the RECON-19 study at the National Institutes of Health where laboratory studies as well as an MRI of the brain and orbit were done that ultimately ruled out neuritis and multiple sclerosis.

**Figure 1:**
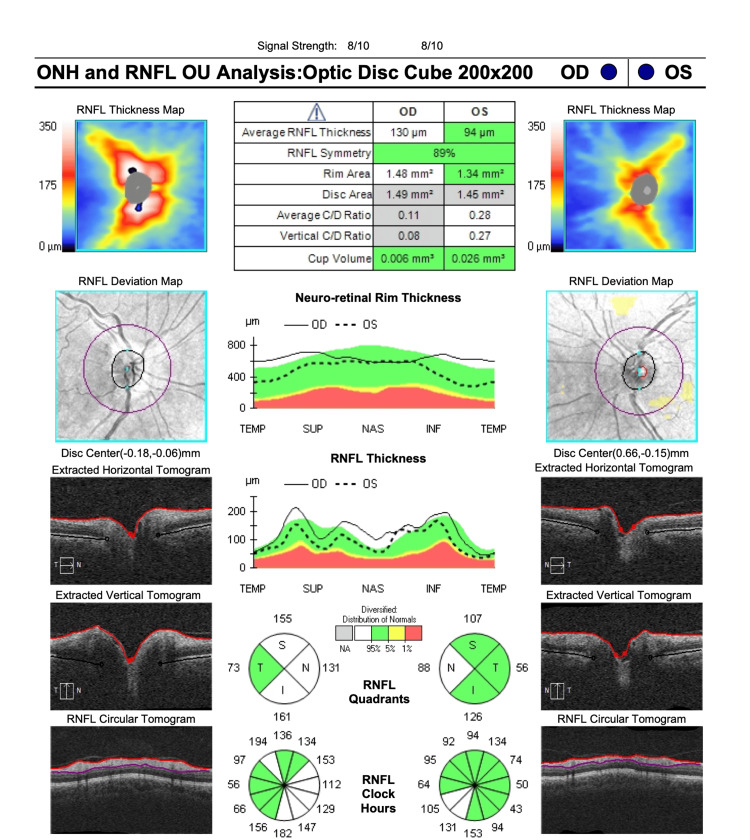
OCT-ONH performed on the patient’s first visit. Scans show swelling of the right optic nerve head according to increased RNFL thickness. OCT-ONH = optical coherence tomography-optic nerve head; RNFL = retinal nerve fiber layer

**Figure 2:**
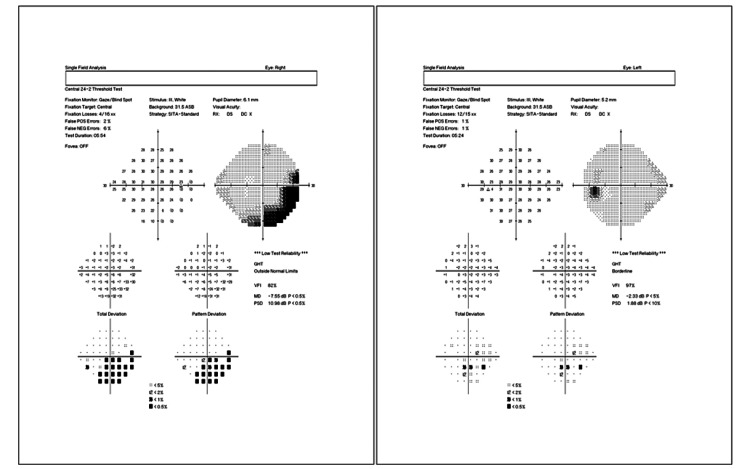
A central 24-2 threshold visual field analysis of the right eye (left image) and left eye (right image) performed on the patient’s first visit showing right visual field loss inferotemporally.

**Figure 3:**
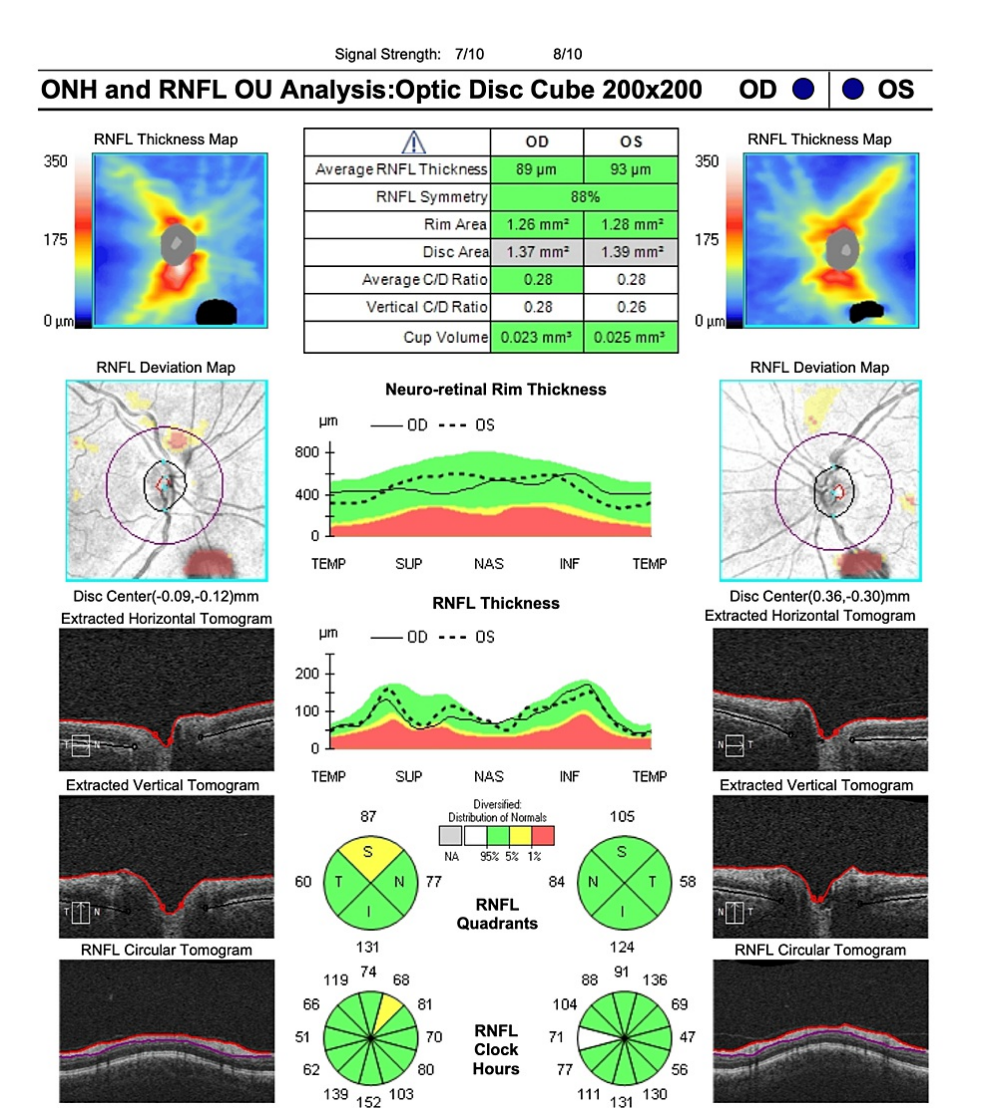
OCT-ONH performed on the patient’s second visit (19 days after the first visit) post intravenous steroid therapy showing improved RNFL thickness in the right eye. OCT-ONH = optical coherence tomography-optic nerve head; RNFL = retinal nerve fiber layer

**Figure 4:**
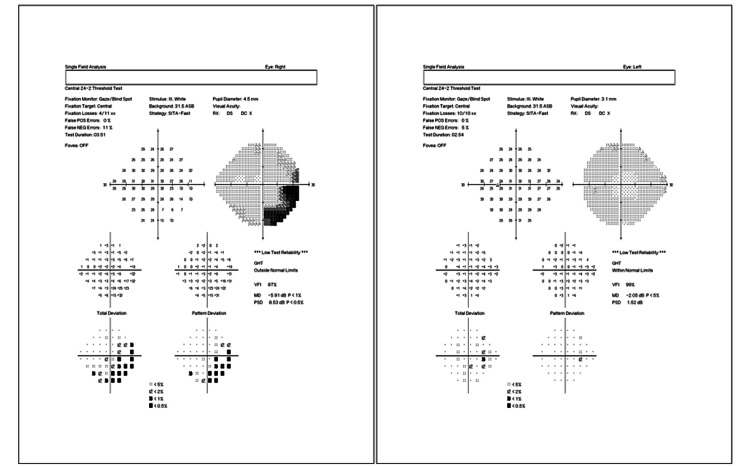
A central 24-2 threshold visual field analysis of the right eye (left image) and left eye (right image) performed on the patient’s second visit (19 days after the first visit) post intravenous steroid therapy. Field analysis shows reduced peripheral vision loss inferotemporally.

**Figure 5:**
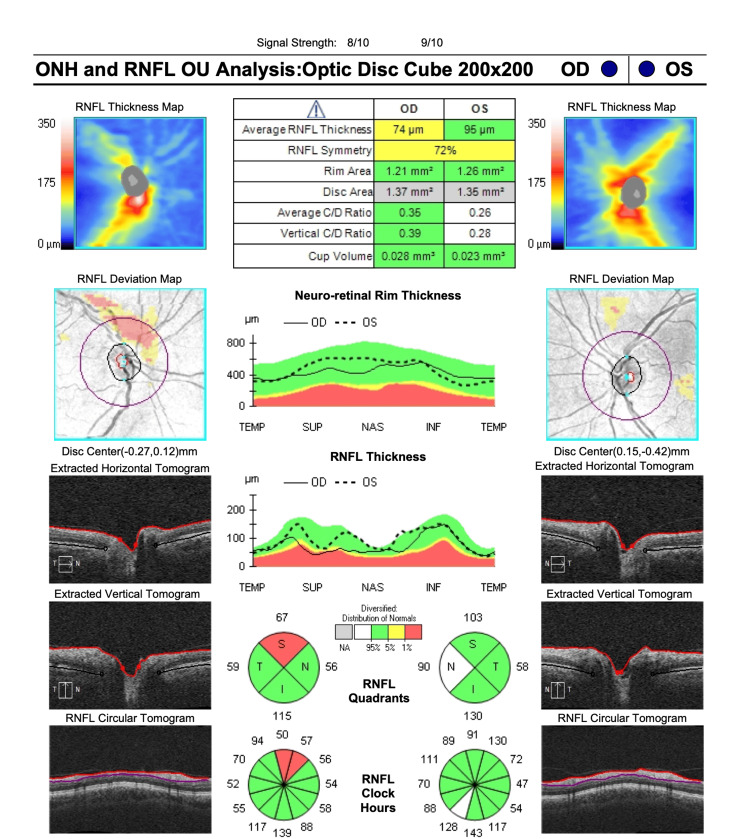
OCT-ONH performed on the patient’s third visit (40 days after the last steroid infusion). OCT shows superior optic nerve atrophy in the right eye based on reduced RNFL. OCT-ONH = optical coherence tomography-optic nerve head; RNFL = retinal nerve fiber layer

**Figure 6:**
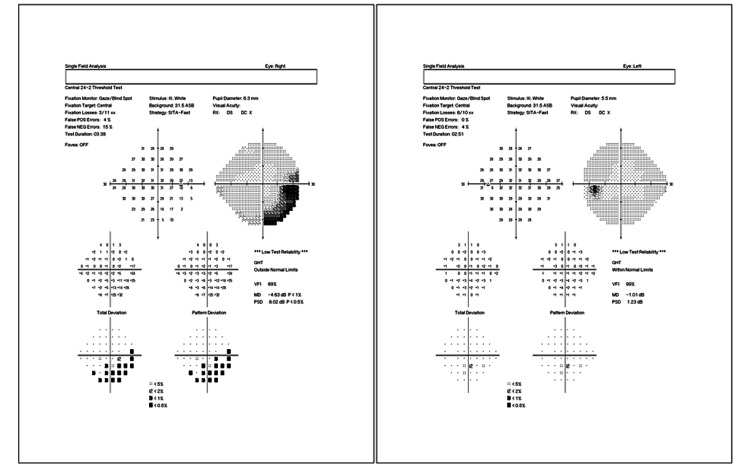
A central 24-2 threshold visual field analysis of the right eye (left image) and left eye (right image) performed on the patient’s third visit (40 days after the last steroid infusion). Field analysis shows a reduction in peripheral vision loss inferotemporally.

At the time of her presentation to our clinic, the initial ophthalmic examination showed visual acuity of 20/20 in both eyes and an intraocular pressure of 15 mmHg and 18 mmHg in the right and left eyes, respectively. Fundus examination, however, showed localized swelling of the optic nerve head in the right eye, which was confirmed with optical coherence tomography (OCT) imaging (Figure 1). A central 24-2 visual field test showed a visual field loss inferotemporally (Figure 2). As a result, the patient was referred to a neurologist for further examinations, where she received five consecutive methylprednisolone infusions intravenously. Nineteen days after the neurology consult, repeat OCT and visual field tests showed a slight improvement in retinal nerve fiber layer thickness (Figures 3, 4). The patient noted a small improvement in vision post-steroid administration, but partial visual field loss remained. A 40-day follow-up after intravenous methylprednisolone showed superior optic nerve atrophy in the right eye (Figure 5). The patient seemed to have sustained a permanent visual field deficit (Figure 6) likely from a COVID-19 hypercoagulable state.

## Discussion

Current literature suggests that the pathogenesis of NAION is multi-factorial, and it is thought to develop due to circulatory insufficiency of the posterior ciliary arteries supplying the optic nerve [1]. This condition is the most common cause of acute optic neuropathy as well as irreversible vision loss in middle-aged and elderly individuals [10,11]. Common risk factors of NAION include hypertension, diabetes, and vasculopathic risk factors, which predispose patients to blood abnormalities such as thrombosis [3].

A literature review confirmed a few cases of NAION in patients with SARS-CoV-2 infection [15]. However, a definite association between NAION and COVID-19 vaccination has not been reported to date [13,16]. Patients with COVID-19 may develop a state of hypercoagulability and hypoxemia, critical aspects that may play a role in the development of NAION, a circulatory insufficiency disease [9]. Studies show that COVID-19 triggers pro-inflammatory cytokines which are established modulators of coagulation and fibrinolysis activation. This increase in pro-inflammatory cytokines can constitute another possible trigger to explain our patient’s coagulopathy [12,17]. Notably, a recent study in patients who recovered from COVID-19 highlighted that SARS-CoV-2 may target the microvascular network of the optic nerve [14].

Our case is atypical because the onset of visual symptoms began months after the initial infection. It is important to note, however, that the patient was a non-smoker with normal bloodwork, no history of hypertension or diabetes, and was not vaccinated for COVID-19. The patient exhibited normal electrocardiogram and pulmonary function test results, as well as a relatively normal brain/orbit MRI. Recent studies discussing thrombotic profiles following COVID-19 vaccination provide insights into understanding how SARS-CoV-2 infection can cause persistent endothelial damage, even in asymptomatic patients or after recovery. These studies confirm that subtle endothelial injury can occur with COVID-19 and remain present beyond the acute infection phase [18,19]. We, therefore, postulate that exposure to COVID-19 may have played a significant role in this pathophysiology as the patient had no other major risk factors for NAION.

Corticosteroid therapy has been a first-line treatment for cases of NAION, leading to recovery of 65% in affected individuals [20]. As seen in our case, the patient did exhibit mild improvement in visual field function and retinal nerve fiber layer thickness. However, complete recovery was not achievable.

This case highlights NAION as a potential sequela of COVID-19 infection, presenting seven months after acute illness in a post-menopausal female without typical risk factors, and serves to raise awareness of ophthalmologic complications that may emerge in the months following recovery from the virus. However, as a single case report, this finding may not be generalizable to the broader population. The role of COVID-19 cannot be definitively established given the lack of blood tests during the acute infection, and further research with larger sample sizes is needed to explore this connection. A noteworthy limitation is that blood tests were done months after the initial infection, thus depriving investigators of insight into the specific ways COVID-19 impacted the patient's health initially. Additional research into the relationship between COVID-19 and the development of ischemic optic neuropathy is warranted to elucidate the implications of this potential association.

## Conclusions

The presented case underscores the potential link between COVID-19 infection and the development of NAION, shedding light on a possible sequela of the virus. This case serves to inform the medical community about ophthalmic symptoms post-COVID-19 infection in unvaccinated individuals and possible effective treatments for NAION. Further research is warranted to explore this connection and enhance our understanding of the disease process.
